# Disruption of the C5a/C5aR1 axis confers protection against hookworm infection in lung

**DOI:** 10.3389/fimmu.2024.1475165

**Published:** 2024-11-19

**Authors:** Sarah Walachowski, Lucien Garo, Arjun Sharma, Archana Jayaraman, Jason Noon, Christoph Reinhardt, Markus Bosmann

**Affiliations:** ^1^ Pulmonary Center, Department of Medicine, Boston University Chobanian and Avedisian School of Medicine, Boston, MA, United States; ^2^ Center for Thrombosis and Hemostasis, University Medical Center of the Johannes Gutenberg-University, Mainz, Germany; ^3^ Mainz Research School of Translational Biomedicine (TransMed), University Medical Center of the Johannes Gutenberg-University, Mainz, Germany

**Keywords:** innate immunity, inflammation, parasitic helminth, myeloid cells, ARDS, ALI

## Abstract

Hookworms are soil-transmitted parasitic nematodes that penetrate the host skin before migrating to the lungs. With an estimated 500-700 million people infected worldwide, hookworm infections are a neglected tropical disease and a significant cause of morbidity, particularly in children, pregnant women, and immunocompromised individuals. Although there is ample evidence that complement activation is pivotal to elicit a protective host immune response against invasive pathogens, its role in hookworm infection remains insufficiently explored. Here, we investigated the complement anaphylatoxin, C5a, during the early lung stage of infection with *Nippostrongylus brasiliensis* in C57BL/6J wild type and C5aR1^-/-^ mice. Despite the previously reported ability of lung larvae to evade complement activation, C5a was detectable locally in lung tissue and bronchoalveolar lavages. Surprisingly, C5aR1 presence directly contributed to the pathogenicity of hookworm infection. The burden of viable parasites in the lungs was mitigated in C5aR1^-/-^ mice, compared to C57BL/6J mice 48 hours post-infection. Additionally, C5aR1^-/-^ mice showed significantly reduced lung injury, lower cytokine release, attenuated alveolar hemorrhage, and limited alveolar-capillary barrier disruption. Neutrophils were the most abundant and highest C5aR1-expressing cell type in the alveolar space after infection. Deficiency of C5aR1 reduced the influx of neutrophils, monocytes, and eosinophils to the pulmonary airways. RNA sequencing of alveolar neutrophils revealed C5aR1-dependent regulation of the novel nuclear protein, DEDD2. In conclusion, our findings highlight the impact of C5aR1 signaling in neutrophils during hookworm infection uncovering an unexpected downside of complement activation in parasitic infection.

## 1 Introduction

Hookworms are soil-transmitted, blood-feeding parasites that infect around 500-700 million individuals worldwide and cause one of the most prevalent neglected tropical diseases (1, 2). Children, pregnant women, and immunocompromised individuals are particularly vulnerable to intestinal blood loss resulting in iron-deficiency anemia and malnutrition (3). This can lead to lower birth weights, cognitive deficits, and indirectly even lower life expectancy (3). Hookworms are generally sensitive to low temperatures and desiccation. Accordingly, infections are most prevalent in hot and moist climates, particularly in areas with poor sanitation and inadequate access to clean water. Infective larvae (stage L3) penetrate the human skin, enter the bloodstream, and are transported to and lodged in the lungs (L4). After breaching the small airways, migrating up the trachea, and being swallowed, the larvae reach the small intestine, where they molt twice (L5) to become mating and egg-laying adults (3). Global control of infection entirely relies on the administration of anthelminthic drugs (donated or low-cost), but high rates of both drug failure (mebendazole) and rapid post-treatment reinfection (within 4-6 months), indicate that overall, current efforts to combat hookworms are inadequate (1, 4, 5). Hookworms have evolved sophisticated mechanisms to withstand host immune responses (6). A deeper understanding of these host-pathogen interactions is essential for devising strategies to control parasites and develop vaccines (1). *Nippostrongylus brasiliensis*, a well-described murine nematode closely related to hookworms that infect humans, has been commonly used to investigate the immune response during the pre-intestinal stage of primary infection (3, 7). During the early pulmonary phase of hookworm infection, larvae face pathophysiological stress and immune challenges while they must navigate complex tissue barriers. However, despite being a potential weak link in the hookworm life cycle, the lung stage of infection remains understudied.

The complement system is a crucial part of innate immunity and plays a key role in defending the lung interface against invading pathogens. It comprises over 50 proteins, either circulating in the blood, deposited intracellularly, or bound to cell membranes (8, 9). The complement factors, including the two major hubs, C3 and C5, are produced in the liver and also locally, particularly in lung cells (10). The most potent anaphylatoxin originating from complement activation is C5a. C5a binds to its two homologous G-protein coupled receptors, C5aR1 (CD88) and C5aR2 (C5L2, GPR77) (11). C5aR1 and C5aR2 are highly expressed on neutrophils, macrophages, monocytes, and some non-myeloid cells (12, 13). While C5aR1 promotes inflammatory responses (degranulation, chemotaxis, cytokine release, and oxidative burst of immune cells), the functional requirement of C5aR2 is more context-/disease-dependent (i.e. anti- or pro-inflammatory activities), or is dispensable in some disease processes (14–19). Excessive C5a generation and signaling through both receptors is linked to severe inflammatory diseases like sepsis and lung injury (20–22).

The magnitude of complement activation during hookworm infection is a complex topic because the parasites may employ counter-strategies tailored to the anatomical location within their hosts (23–25). For example, substantive deposition of C3 and adherence of eosinophil-rich leukocytes was reported with infective-stage (L3) but not with lung-stage (L4) larvae of *N. brasiliensis* (24). Furthermore, *N. brasiliensis* infected-C3^-/-^ mice showed only slightly higher (10-15%) lung parasite counts compared to wild type mice (26). However, inbred FVB/N mice that are deficient in C5 are highly resistant to *N. brasiliensis* infection (27), although it remains unclear whether this is due to other gene mutations in this strain (Wong).

Here, we investigated the role of C5a/C5aR1 signaling in the host response during the early intrapulmonary life cycle of *N. brasiliensis* infection. Surprisingly, C5aR1 deficiency was associated with reduced alveolar parasite viability, less neutrophil influx, decreased lung injury, and a lower inflammatory response. Transcriptomic analyses of alveolar neutrophils revealed *Dedd2* as a novel C5aR1-regulated gene that has been implicated in cell death pathways (28, 29).

## 2 Methods

### 2.1 Mice

All procedures with mice were approved by the Boston University Institutional Animal Care and Use Committee and performed in compliance with the guidelines of the National Institutes of Health. C57BL/6J mice were obtained from The Jackson Laboratory (Bar Harbor, ME). C5aR1^-/-^ (B6.129S4-C5ar1tm1Cge) were originally provided by Prof. J. Köhl, Lübeck University. C5aR2^-/-^ (C5ar2tm1b(EUCOMM)Wtsi) from targeted embryonic stem cells and C5aR1/2^-/-^ (C57BL/6J-Del(7C5ar2-C5ar1)1Bosm) mice were generated in our laboratory and the Transgenic Facility of the University of Mainz, Germany (submitted for publication elsewhere). The mice were bred and genotyped at the animal facilities of Boston University under specific pathogen-free conditions and a controlled light/dark cycle. Male and female mice at 8-12 weeks of age were used for experiments.

### 2.2 *Nippostrongylus brasiliensis* cultures and inoculations

*N. brasiliensis* infective L3 larvae (3-6 mm length) were visualized through an Amscope binocular (4 x magnification) and recorded using a camera with 10 x magnification (**Supplementary Movie 1**). Larvae were maintained and separated from coprocultures, and ultimately prepared for inoculations according to an established protocol (7). Mice received subcutaneous (s.c.) injection in the flank with n=500 L3 in 0.1 mL of sterile PBS (Thermo Fisher Scientific, Waltham, MA). Sham mice treated with sterile PBS s.c. were included as controls.

### 2.3 Alveolar and lung tissue parasite burdens

Alveolar and lung viable larvae burdens were assessed in bronchoalveolar lavage fluids (BALFs) and lung tissues respectively, isolated from mice 48 h post-infection following a previously established protocol (30).

### 2.4 Isolation and culture of macrophages

Alveolar macrophages were isolated from naïve C57BL/6J mice by performing BAL using 6x1 mL aliquots of cold sterile phosphate buffered saline (PBS) supplemented with 0.1% BSA (Sigma) and 2 mM ethylenediaminetetraacetic acid (EDTA) (Invitrogen, San Diego, USA) by inserting an 18G angiocatheter (Exel International, Redondo Beach, USA) into the trachea and kept on ice. The lavages were combined and centrifuged at 650 g for 10 min at 4°C. BALF cells were resuspended in sterile Roswell Park Memorial Institute medium (RPMI)-1640 (Gibco, Life Technologies, Grand Island, USA) medium containing 0.1% bovine serum albumin (BSA) and 100 U/mL Penicillin-Streptomycin (Thermo Fisher Scientific), and counted with a cell counter (CytoSMART, Skillman, USA). Equal numbers of cells were seeded in 48-well plates (Corning, Corning, USA) and cells were cultivated for 2 h in 5% CO_2_ humidified incubator at 37°C. Thereafter, non-adherent cells were removed by washing the wells once with sterile PBS and the attached cells were further stimulated with 100 mM recombinant mouse C5a (rmC5a, R&D systems, Minneapolis, USA).

### 2.5 Lung injury quantification

BALF total protein was measured with the Pierce BCA Protein Assay Kit (Thermo Fisher Scientific). Alveolar hemorrhage was assessed as described elsewhere (30). Briefly, a serial dilution (8000-250 mg/mL) of human hemoglobin (Sigma-Aldrich, St. Louis, MO) was prepared, and 50 μL of each standard and BALF samples were added to duplicate wells of a 96-well plate. A volume of 100 μL of 6% SDS (Sigma-Aldrich) was added to all wells and resuspended several times. The absorbance at 560 nm was measured with a Tecan Infinite M Nano plate reader (Tecan, Mannedorf, Switzerland), and alveolar hemorrhage (i.e., total amount of hemoglobin recovered in BALF) was determined from the standard curve generated by Magellan version 7.2 software (Tecan).

### 2.6 ELISA

The ELISA kits for IL-27p28, IL-6, CXCL10, TNF-α were purchased from R&D System (Minneapolis, MN). BALF samples were diluted in PBS with 0.1% BSA to fit in standard range of the ELISA kit and measured according to the ELISA kit protocol. The absorbance at 450 nm was measured with a Tecan Infinite M Nano plate reader, and concentration was determined from the standard curve generated by Magellan software. The manufacturer’s protocol was followed for analyses. For detection of citrullinated histones, an ELISA kit for total histone detection was purchased from Roche (Mannheim, Germany). Anti-citrullinated Histone H3 antibody (Abcam) was used as coating antibody instead of anti-histone biotin. Optical density was read at 450 nm or 405 nm. This modified citH3 ELISA produced minimal background signal when sham mouse BALF were used as negative control (31, 32).

### 2.7 Flow cytometry

BALF samples were collected from infected mice as described above and kept on ice. Cell viability from the single cell suspension (>98%) was confirmed by staining with fixable viability dye eFluor 780. BALF cells were centrifuged at 650 g, 4°C for 5 min, and incubated at 4°C in the dark for 10 min with Fc receptor block (anti CD16/CD32 antibody) followed by fluorophore-labelled mouse antibodies, listed in the **Supplementary Table**. All antibodies were diluted in fluorescence-activated cell sorting (FACS) buffer containing sterile PBS supplemented with 1% (w/v) BSA, 0.01% (w/v) sodium azide, and 2.5 mM EDTA. The stained cells were washed twice in FACS buffer and fixed in 2% paraformaldehyde (Santa Cruz Biotechnology, Dallas, TX) at 4°C for 20 min in the dark.

For intranuclear staining of DEDD2 and intracytoplasmic staining of THBS1, cells were fixed and permeabilized after surface marker staining using the eBioscience FoxP3/TF staining buffer set (Thermo Fisher Scientific) for DEDD2, or the Cytofix/Cytoperm kit (BD Biosciences) for THBS1. Cells were further incubated with unconjugated anti-mouse DEDD2 polyclonal antibody (or isotype control) for 45 min at 4°C, or with unconjugated anti-mouse THBS1 monoclonal antibody (or isotype control) for 20 min at 4°C. After two washing steps, cells were stained for another 30 min at 4°C with the corresponding fluorophore-labelled secondary antibodies.

**Figure 1:**
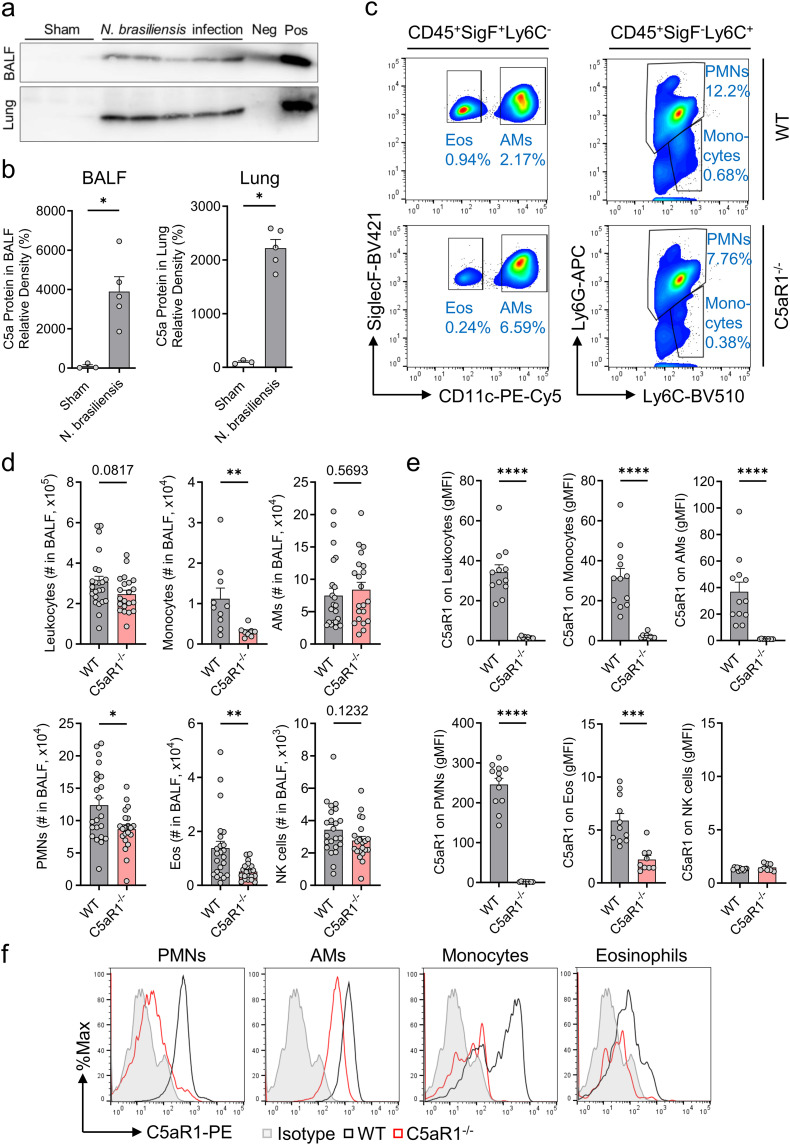
C5aR1 signaling contributes to immune cell influx to airways during primary *N. brasiliensis* infection. **(A–F)** *In vivo* comparison of the susceptibility of C5aR1^-/-^ mice and WT control (C57BL/6J) mice to primary *N. brasiliensis* infection (500 L3 per mouse, subcutaneously) in the lungs 48 h post-infection. **(A)** Western blots of immunoprecipitated C5a from BALF and lung lysates collected from uninfected (Sham, n=3) or infected WT mice (n=5). Positive (pos, rmC5a, 10 ng) and negative controls (neg, immunoprecipitation medium containing Protein A agarose beads and Laemmli buffer without rmC5a) were added to the gels. **(B)** Densitometry of these bands reflecting C5a abundance in BALF and lung lysate. Samples were normalized to uninfected WT mice (Sham). **(C)** Representative flow cytometry plots of CD45^+^SiglecF^+^Ly6C^-^ cells (left panels) and CD45^+^SiglecF^-^Ly6C^+^ cells (right panels) from BALF of WT control (top panels) and C5aR1^-/-^ mice (bottom panels). Cell frequencies of live populations are indicated next to each gate. **(D)** Absolute numbers (#) of CD45^+^ leukocytes, Ly6G^-^Ly6C^+^ monocytes, SiglecF^+^CD11c^+^ AMs, Ly6G^+^Ly6C^+^ PMNs, SiglecF^+^CD11c^-^ Eos, and CD49b^+^ NK cells isolated from BALF and determined by flow cytometry (n≥20/group). **(E)** C5aR1 surface expression on each BALF cell population expressed in gMFI (n≥8/group). **(F)** Representative histograms of C5aR1 expression on PMNs, AMs, monocytes, and Eos. Isotype controls for all antibodies were pooled and total live alveolar cells are shown. Data illustrated in **(A, B)** are representative of 2 independent experiments. **(D)** shows pooled values from 2 independent experiments. **(E, F)** are representative of 3 independent experiments. Each circle represents an individual mouse. Data are shown as mean ± SEM and were analyzed by Student’s t-test.*P<0.05; **P<0.01; ***P<0.001; ****P<0.0001. BALF, bronchoalveolar lavage fluid. PMNs, polymorphonuclear leukocytes or neutrophils. AMs, alveolar macrophages. Eos, eosinophils. NK, natural killer cells. gMFI, geometric mean of fluorescence intensity. rmC5a, recombinant mouse C5a.

Stained/fixed cells were washed twice and centrifuged as described before, resuspended in FACS buffer, and transferred to a FACS tube containing 20 μL of CountBright Absolute Counting Beads (Thermo Fisher Scientific). For all single-stained compensation controls, we used a pool of BALF cells or UltraComp eBeads Compensation Beads (Invitrogen, Waltham, MA) according to the manufacturer’s instructions. Multiplets were identified as FSC-W^hi^ (i.e., FSC-A^hi^) cells and excluded from further analysis (33). For **Figure 1F**, isotype controls for all antibodies were pooled and total live alveolar cells are shown. Flow cytometric acquisition of samples was performed on a BD LSR II flow cytometer with BD FACSDiva software or an Astrios MoFlo instrument (Beckman and Coulter). Preparation and analyses of final plots were performed in FlowJo version 10.

### 2.8 Immunodetection of C5a

C5a protein detection was performed via tandem immunoprecipitation followed by western blotting of lung and BALF samples. This previously described protocol was intended to optimize sensitivity and specificity (34, 35). The faint signal in the BALF negative control without sample lane was interpreted as spillover from the recombinant mouse C5a (rmC5a) positive control lane (**Figure 1A**).

### 2.9 Reverse transcription and real-time PCR

Total RNA was isolated from macrophages by Qiazol/chloroform/isopropanol/EtOH purification or the innuPREP RNA Mini kit (AnalytikJena, Jena, Germany), and quantified using a NanoDrop 2000c spectrophotometer (Thermo Fisher Scientific). The cDNA was generated using the Maxima™ H Minus cDNA Synthesis Master Mix kit (Thermo Fisher Scientific). 3-5 ng cDNA complemented with PowerUp™ SYBR™ Green Master Mix (Applied Biosystems, Waltham, USA) and specific primers at a concentration of 500 nM each were used for RT-PCR in a QuantStudio 3 Real-Time PCR instrument (Applied Biosystems). *Dedd2* gene expression was compared between samples by normalizing to *Gapdh* expression and applying the 2^-ΔΔCt^ formula. Primer sequences are provided in the **Supplementary Table**.

### 2.10 RNA-seq

RNA was extracted using the QIAGEN RNAeasy Plus Micro kit. Ultra-low input RNA-seq was performed by Novogene (USA). Analysis methods are further detailed in the **Supplementary Material**. *C5ar1* was not included in the volcano plot for better axis scaling, but absence of expression was confirmed in all C5aR1^-/-^ mice. Pathways with no apparent biological relevance to pulmonary hookworm infection (e.g. pancreas beta cells, estrogen early response, allograft rejection, adipogenesis, xenobiotic metabolism and heme metabolism) are not shown.

### 2.11 Statistical analyses

Statistical analyses were performed with Prism v8 software (GraphPad). Sample sizes and number of technical and biological replicates are included in the figure legends. Data in the bar graphs represent mean ± standard error of the mean (SEM). Comparisons of two groups were performed based on the two-sided Student’s *t*-test while multiple group testing was done through one-way analysis of variance (ANOVA) with Bonferroni correction. The statistical significance threshold was set at P<0.05.

## 3 Results

### 3.1 C5aR1 signaling induces immune cell influx to airways during primary *N. brasiliensis* infection

To determine if pulmonary hookworm infection triggers local C5a generation as an indicator of early complement activation, we used *N. brasiliensis*, a well-characterized murine nematode closely related to human-pathogenic hookworms. This infection model is well established for studying the immunological response during the pre-intestinal stage of primary infection (3, 7).

C57BL/6J wild type (WT) mice were inoculated sub-cutaneously (s.c.) with viable stage 3 N*. brasiliensis* larvae (L3) from coprocultures (**Supplementary Movie 1**). Bronchoalveolar lavage (BALF) and lung tissue were collected 48 h post-infection and processed to detect C5a by immunoprecipitation followed by western blot. Infected mice displayed a considerable presence of C5a in BALF and dissociated lungs, whereas C5a was not detected in uninfected sham mice (**Figures 1A, B**). Next, we proceeded with immunophenotyping of BALF immune cells isolated from WT and C5aR1^-/-^ mice using flow cytometry (gating strategy provided in **Supplementary Figure 1A**). *N. brasiliensis* caused a marked accumulation of PMNs (neutrophils), monocytes, and eosinophils into the airspaces in the lungs in both strains (**Figures 1C, D**), which are not present in the BALF of uninfected sham mice (**Supplementary Figure 1B**) (35, 36). Importantly, the influx of PMNs, monocytes, and eosinophils was significantly reduced in C5aR1^-/-^ mice compared to WT mice, suggesting that the recruitment of inflammatory cells to the alveoli during the early phase of hookworm infection is dependent on the C5a/C5aR1 axis.

In contrast, the numbers of C5aR1^+^ resident AMs (alveolar macrophages) and invading NK cells remained unchanged in the absence of C5aR1 signaling, since these cells do not require C5a/C5aR1 to appear in the small airways of the lung (**Figures 1D–F**). All studied subsets of BALF leukocytes except NK cells expressed C5aR1 (**Figures 1E, F**). PMNs, not eosinophils, were the most abundant cell type, and PMNs expressed highest amounts of C5aR1 (**Figures 1D–F**). These data suggest that C5a mediates innate immune responses, particularly PMN influx, during acute pulmonary hookworm infection.

### 3.2 C5aR1 deficiency reduces parasite burden and attenuates the severity of lung injury

**Figure 2:**
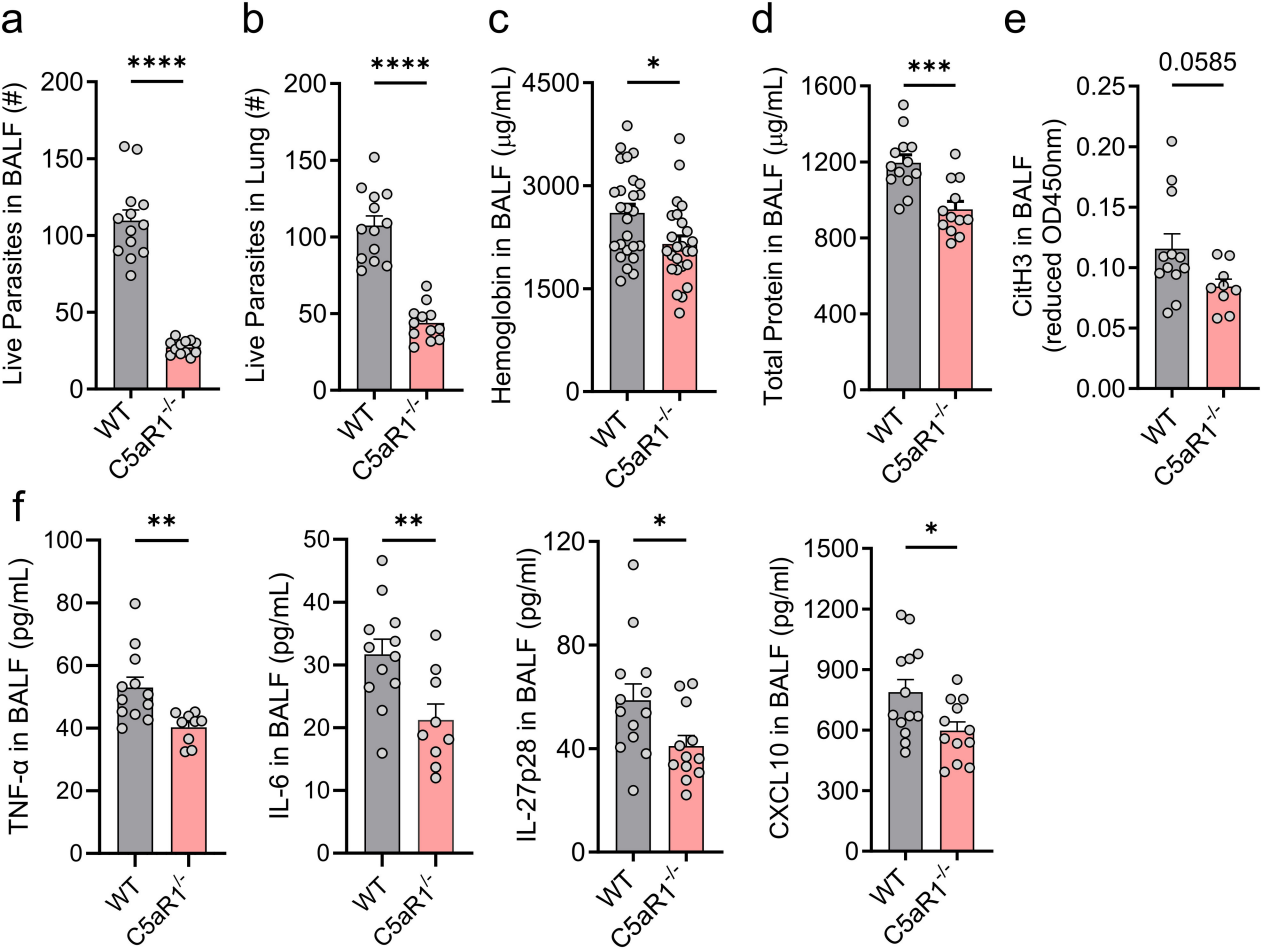
C5aR1 deficiency reduces parasite burden and attenuates the severity of lung injury and inflammation. **(A–F)** C5aR1^-/-^ mice and WT control mice were primarily infected with *N. brasiliensis* (500 L3 per mouse, subcutaneously), and lungs and BALF were collected 48 h post-infection. Parasite burden in **(A)** BALF and **(B)** within the lung tissue. # represents the absolute number of worms (n≥12/group). **(C)** Hemoglobin as a marker of alveolar hemorrhage was quantified in fresh BALF samples by colorimetric assay (n≥25/group). **(D)** Total protein concentration, a measure of alveolar-capillary barrier disruption, was determined using a BCA assay (n≥12/group). **(E)** CitH3, a marker of NETosis (n≥9/group) and **(F)** the inflammatory cytokines, TNF-α, IL-6, IL-27p28 and CXCL10, were quantified in cell-free supernatants by ELISA (n≥9/group). Data illustrated in **(A, B, D–F)** are representative of 3 independent experiments; panel **(C)** shows pooled values from 2 independent experiments. Each circle represents an individual mouse. Data are shown as mean ± SEM and were analyzed by Student’s t-test. *P<0.05; **P<0.01; ***P<0.001. BALF, bronchoalveolar lavage fluid. CitH3, citrullinated histone 3.

PMNs are a first line of defense and have the potential to kill nematode larvae (37, 38). Consequently, BALF and lung lysates from WT and C5aR1^-/-^ mice were used to evaluate the impact of C5aR1 deficiency on alveolar and lung parasite burden. Surprisingly, WT mice compared to C5aR1^-/-^ mice showed a significant ~4-fold and ~2-fold higher viable parasite load in BALF and lung homogenates, respectively (**Figures 2A, B**). To assess a potential role of the C5aR2 receptor, we compared WT mice with C5aR1^-/-^, C5aR2^-/-^, and C5aR1/2 dual knockout mice. No relevant changes in the alveolar live parasite counts in infected C5aR2^-/-^ and C5aR1/2^-/-^ mice were present (**Supplementary Figure 2A**). These results indicated a more efficient pathogen clearance in alveolar spaces and lung tissue when C5a signaling through C5aR1 was absent, despite a reduced PMN count. Since hookworms cause vascular damage in lung tissue, we assessed the alveolar hemorrhage by measuring hemoglobin concentration in the BALF of infected WT and C5aR1^-/-^ mice. In line with greater parasite burden, we observed greater alveolar hemorrhage in WT mice compared to C5aR1^-/-^ mice (**Figure 2C**). On the other hand, the disruption of C5aR2 signaling alone or in combination with C5aR1 did not significantly alter pulmonary hemorrhage (**Supplementary Figure 2B**). Consistently, lung injury was more severe in WT mice than in the C5aR1^-/-^ mice, as shown by the exacerbated protein leakage detected in BALF (**Figure 2D**), a parameter used as a marker of capillary/alveoli barrier disruption (39). Furthermore, PMN reduction in C5aR1^-/-^ mice was associated with a lower concentration of soluble citrullinated histone 3 (CitH3), suggesting a dampened release of neutrophil extracellular traps (NET)s, consistent with the reduction of tissue damage observed in C5aR1^-/-^ mice (**Figure 2E**). CitH3 is regarded as a reliable marker of NETosis (31, 40). For this reason, we opted not to analyze additional parameters of neutrophil activation. To evaluate the intensity of parasite-induced inflammation, we determined a range of inflammatory markers in BALF of WT and C5aR1^-/-^ mice early after *N. brasiliensis* infection. Significantly lower levels of TNF-α, IL-6, IL-27p28 and CXCL10 were found in the BALF of C5aR1^-/-^ mice, showing that abrogation of C5aR1 signaling attenuated inflammatory cytokines and chemokines (**Figure 2F**). Of note, we determined that the surge of BALF larvae burden and lung injury/inflammation occurs 2 days after infection compared to uninfected sham controls (**Supplementary Figures 3A-D**).

### 3.3 Transcriptomic profiling of neutrophils from C5aR1^-/-^ mice after infection with *N. brasiliensis*

**Figure 3:**
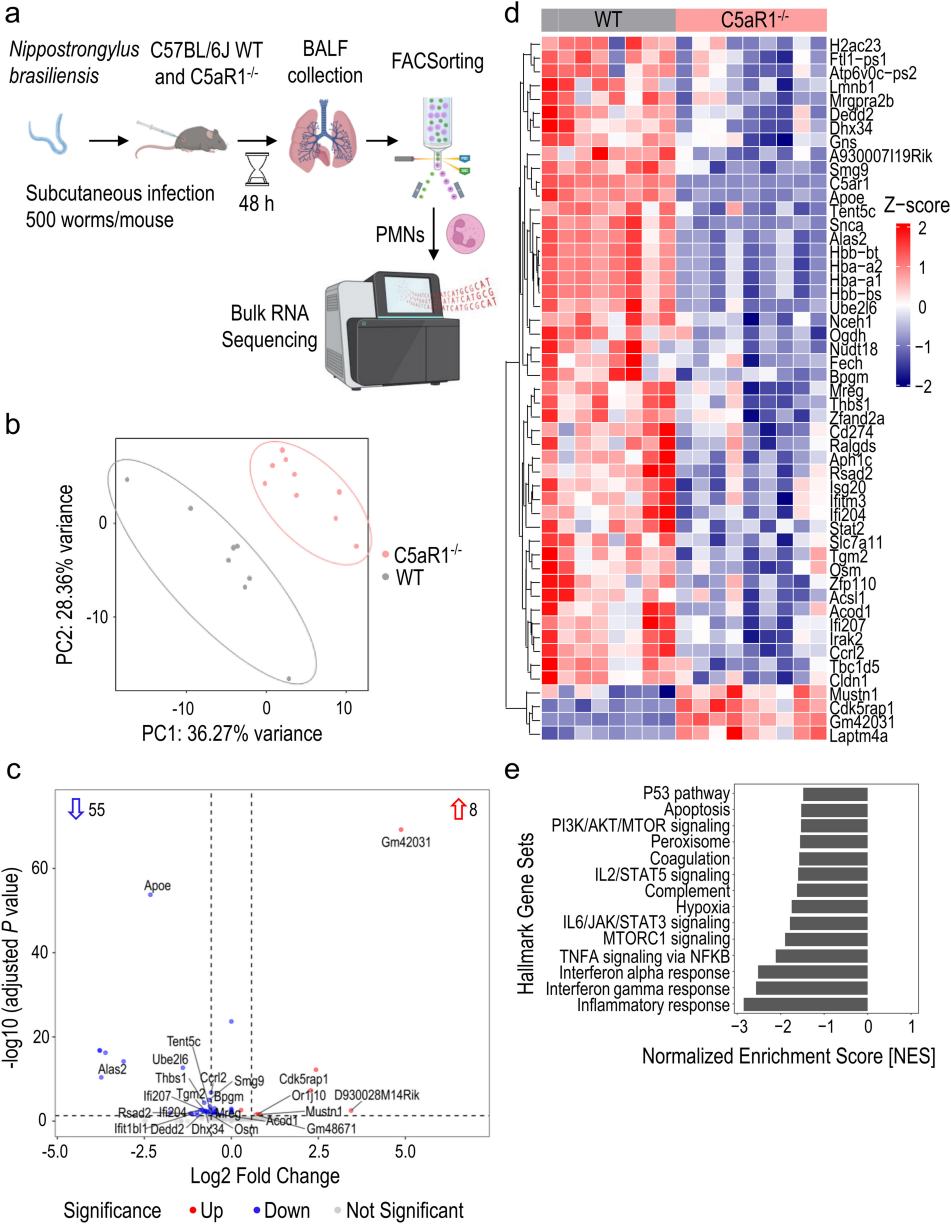
Transcriptomic analysis reveals a diminished inflammatory response in neutrophils from C5aR1^-/-^ mice after infection with *N. brasiliensis*. **(A)** Schematic workflow of the bulk RNA-seq experiment. **(B)** Principal component analysis plot showing distinct separation between WT and C5aR1^-/-^ neutrophils based on the top 500 variable genes. **(C)** Volcano plot of the bulk RNA-seq data. The numbers within the plot indicate the statistically significant up-/downregulated genes. The horizontal dashed line represents the threshold of adjusted P<0.05. The vertical dashed lines indicate the threshold of absolute log2 fold change>0.58. C5aR1 is not shown for better axis scaling. **(D)** Heatmap of the expression profiles of statistically significant up-/downregulated genes. Normalized expression (z-scores; −2/blue to +2/red) of genes with a baseMean≥25 were plotted. **(E)** Bar plot showing significant hallmark gene sets from enrichment analysis. n=8 in WT group and n=9 in C5aR1^-/-^ group. Statistical significance: adjusted P<0.05. PMNs, polymorphonuclear leukocytes or neutrophils.

Since PMNs were the most abundant cell type with the highest C5aR1 expression, and C5aR1^-/-^ mice showed significantly lower neutrophil recruitment to the alveolar compartment than WT mice, we investigated C5aR1-dependent alterations in PMNs using bulk RNA sequencing. RNA was purified from fluorescence activated cell (FAC)Sorted CD45^+^Ly6C^+^Ly6G^+^ PMNs isolated from the BALF of C5aR1^-/-^ and WT mice infected with *N. brasiliensis* for 48 h (**Figure 3A**). The principal component analysis plot showed 2 distinct clusters of samples respective of each experimental group (**Figure 3B**). *N. brasiliensis* infection induced significant changes in the abundance of 63 genes in C5aR1^-/-^ versus WT mice, with a large majority of differentially expressed genes (DEGs) being downregulated (n=55) and only a few DEGs being upregulated (n=8) (**Figure 3C**). Among DEGs with the highest expression values, we identified *Dedd2*, *Thbs1* (*Thrombospondin1*), *Ccrl2*, and *Cd274* (encoding for PD-L1), that were downregulated in C5aR1^-/-^ PMNs (**Figure 3D**). Enrichment analysis of the DEGs showed that apoptosis pathway/P53 signaling, inflammatory response represented by PI3K/AKT/MTOR signaling, IL2/STAT5 signaling, IL6/JAK/STAT3 signaling, MTOR signaling, peroxisome, TNF-α signaling and hypoxia, plus interferon α/β pathways, were significantly inhibited in PMNs during *N. brasiliensis* infection (**Figure 3E**). The number of genes involved in the regulation of the innate immune response, defense, and metabolic processes were homogenously distributed (**Supplementary Figure 4**). In conclusion, these results suggest that C5aR1^-/-^ PMNs harbor a less inflammatory phenotype than WT PMNs.

### 3.4 C5aR1 signaling regulates DEDD2 in alveolar neutrophils during *N. brasiliensis* primary infection

**Figure 4:**
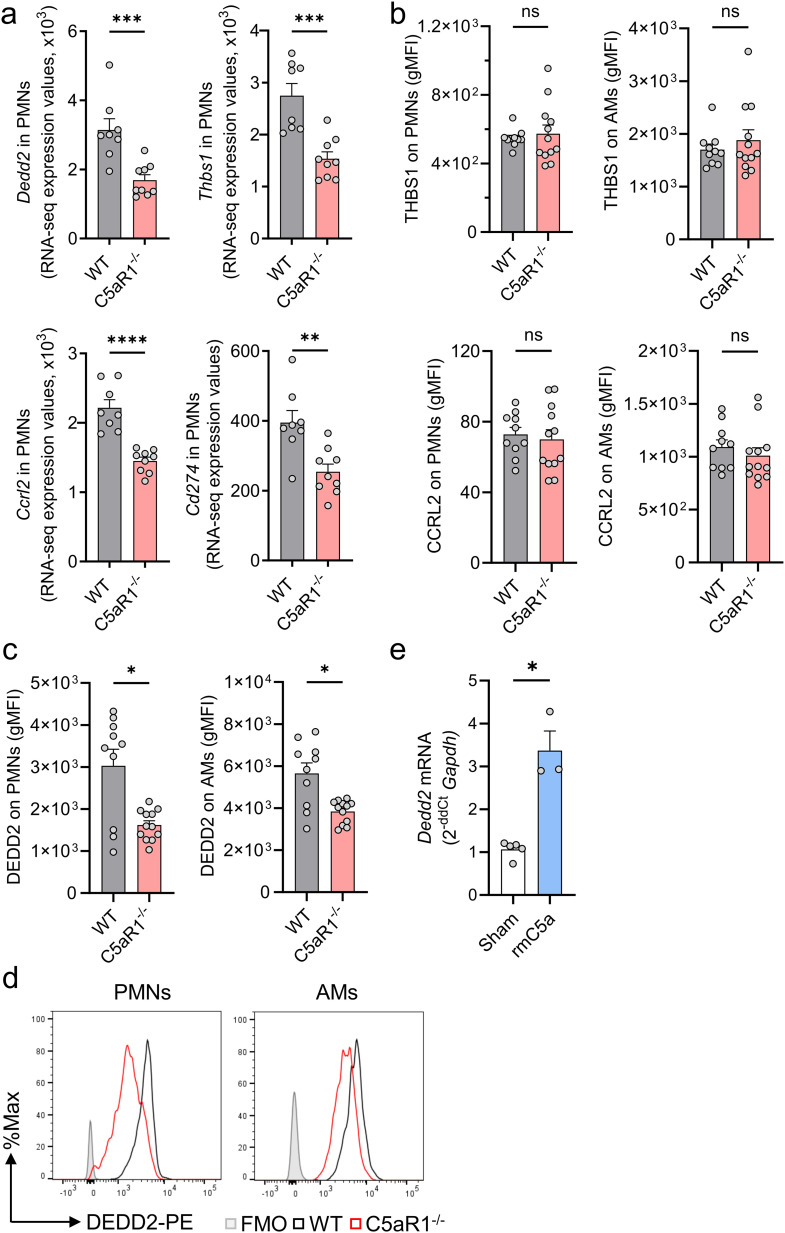
C5aR1 regulates DEDD2 in alveolar macrophages and neutrophils during *N. brasiliensis* primary infection. **(A–D)** C5aR1^-/-^ and WT mice were infected with *N. brasiliensis* (500 L3 per mouse, subcutaneously) and BALF was collected after 48 (h) **(A)** Comparison of selected DEGs in WT and C5aR1^-/-^ PMNs during *N. brasiliensis* infection. *Dedd2*, *Thbs1*, *Ccrl2*, and *Cd274* genes were shortlisted according to their level of expression (baseMean>300), log2 fold change<-0.53 and adjusted P<0.05. Expression values obtained from the bulk RNA sequencing experiment (normalized counts) were plotted for each biological replicate. n=8 in WT group and n=9 in C5aR1^-/-^ group. **(B)** THBS1, CCRL2, and **(C, D)** DEDD2 protein were assessed in alveolar PMNs and AMs by flow cytometry. gMFIs representative of 2 independent experiments were plotted (n=10 in WT group and n=12 in C5aR1^-/-^ group) and representative histograms of DEDD2 expression in PMNs and AMs are shown. Filled grey line indicates FMO control. **(E)** *Dedd2* gene expression in AMs from naïve WT mice (n=4) with or without stimulation for 6 h with 100 mM rmC5a was determined by RT-qPCR. Data were normalized to *Gapdh* expression and are shown as fold change compared to unstimulated controls (Sham). **(A-C)** Each circle represents an individual mouse. **(E)** Each data point is the mean ± SEM of technical triplicates of one representative experiment. Data are shown as mean ± SEM and were analyzed by Student’s t-test.*P<0.05; **P<0.01; ***P<0.001, ****P<0.0001. DEGs, differentially expressed genes. DEDD2, death effector domain containing 2.; THBS1, Thrombospondin 1; BALF, bronchoalveolar lavage fluid; PMNs, polymorphonuclear leukocytes or neutrophils; AMs, alveolar macrophages; FMO, Fluorescence Minus One; gMFI, geometric mean of fluorescence intensity; rmC5a, recombinant mouse C5a. ns, not significant.

We manually curated a short list of significant DEGs of interest based on perceived biological relevance, novelty, and abundance. The expression values of *Dedd2*, *Thbs1*, *Ccrl2* and *Cd274* were significantly reduced in C5aR1^-/-^ alveolar PMNs after *N. brasiliensis* infection (**Figure 4A**). Since *Dedd2*, *Thbs1*, and *Ccrl2* genes (but not *Cd274*) showed high expression values, we proceeded with protein validation by flow cytometry. No significant difference was observed in THBS1 or CCRL2 protein expression in Ly6G^+^Ly6C^+^ PMNs and CD11c^+^SiglecF^+^ AMs between C5aR1^-/-^ mice and WT controls (**Figure 4B**). However, protein staining for DEDD2 was reduced by 2-fold in PMNs and by 1.5-fold in AMs of C5aR1^-/-^ mice compared with WT mice following *N. brasiliensis* infection (**Figures 4C, D**). These findings confirmed the downregulation of DEDD2 at the protein level in neutrophils lacking C5aR1 during hookworm infection, while suggesting that potential post-transcriptional regulation mechanisms may occur for CCRL2 and THBS1. DEDD2 is a recently identified protein with potential functions as a cell death regulator and possibly apoptotic processes (28) (**Figure 2E**). To further evaluate the effect of C5a signaling through C5aR1 on *Dedd2* expression, we exposed primary AMs isolated from naïve WT mice to mouse recombinant C5a and assessed the gene expression of *Dedd2*. Consistent with the *in vivo* RNA sequencing data (**Figure 4A**), C5a stimulation resulted in upregulation of *Dedd2* in AMs (**Figure 4E**), demonstrating the C5a-dependent regulation of DEDD2 through C5aR1.

## 4 Discussion

This study provides new insights that C5a plays a detrimental role during the early lung phase of infection with *N. brasiliensis*. The release of anaphylatoxin C5a in lungs and bronchoalveolar spaces resulted in a robust influx of C5aR1-expressing neutrophils and monocytes. The absence of C5aR1 signaling decreased the local accumulation of inflammatory cells and the inflammatory milieu. C5aR1-deficient mice showed less severe injury than the WT mice, suggesting that C5aR1 abrogation attenuated alveolar inflammation and partially rescued barrier dysfunction early after *N. brasiliensis* infection. Additionally, transcriptomic analyses of alveolar neutrophils identified a small subset of C5a/C5aR1-regulated genes such as DEDD2, a novel caspase-8/-10 regulator (28, 29).

A few studies have reported the pivotal function of the alternative pathway in response to *N. brasiliensis in vitro* and *in vivo*, especially for mouse C3 deposition and leukocyte adherence on L3 infective-stage larvae (23–25). Indeed, serum addition in co-cultures of neutrophils and monocytes with larvae for 24 h increased adherence, suggesting that leukocyte recognition of larvae was a complement-mediated process (23). Strikingly, human C3 deposition to larvae largely depended on the classical or lectin pathways but not on the alternative pathway as did mouse C3 (25). Interestingly, C3-, C1q-, or factor B-deficiency had no effect on the parasite burden in the lungs 24 h post-infection in mice, whereas C3- and factor B-deficient mice showed slightly higher lung burden than WT mice after 48 h of *N. brasiliensis* infection (26). A consistent finding with other infectious models such as bacterial pneumonia has been that mice lacking C3 (and factor B) exhibit an increased susceptibility and more pronounced acute lung injury than WT counterparts (41). Notably, primary infected FVB/N mice, which are deficient in complement protein C5, displayed a less severe lung infection which led to a reduced larvae burden downstream in the gut (27). Because FVB/N mice displayed no significant difference in skin inflammatory responses or larvae burden to *N. brasiliensis*, and FBV/N mice displayed no resistance to *H. bakeri*, a gut parasite lacking a lung transitory stage, the authors interpreted FVB/N resistance to involve pulmonary immunity. Unlike infection in our C5aR1^-/-^ mice, *N. brasiliensis* resistance described in FVB/N hosts could not be definitively attributed to deletion of C5, as this mouse strain possessed numerous other gene mutations (42). While broad complement deficiency did not drastically affect lung larvae burden (26), our study evidenced a drastic reduction of viable parasites count in BALF in C5aR1^-/-^ mice, but not in C5aR2^-/-^ or in C5aR1/2^-/-^ double knockout mice. Consequently, our findings indicate that the absence of C5aR1 signaling confers protection against *N. brasiliensis* infection. In other models of inflammation and infection, C5a also aggravates lung injury (43) and the disruption of endogenous C5a signaling is strongly protective (18, 35, 44, 45). DEDD2, which was previously described as a potent apoptosis inducer through a Casp-8/-10-dependent mechanism (29), appears to be regulated by C5aR1 during hookworm infection. Indeed, we observed the downregulation of DEDD2 expression at the RNA and protein level in C5aR1^-/-^ macrophages and neutrophils after *N. brasiliensis* infection. Interestingly, C5a-mediated lung injury following ischemia/reperfusion was exacerbated by induced apoptosis in alveolar macrophages (43). Due to the non-existence of appropriate tools such as DEDD2-deficient mouse strains or DEDD2-blocking antibodies, we could not further investigate the biological relevance of DEDD2 during the early pulmonary stage of *N. brasiliensis* infection.

Our data implicate a role for dysregulated neutrophil responses in the protection of C5aR1^-/-^ mice during pulmonary hookworm infection. However, the importance of T-helper 2-type responses, including IgE production, a distinct panel of cytokines (IL-4, IL-5, IL-13), and the concomitant mobilization of specific effector cells such as eosinophils, basophils, and mast cells, is undisputed during parasite clearance (46–50). These subsequent adaptive immune responses are unlikely to play a major role in host defense against hookworm infection in the lung during the first 2-3 days of primary infection. While we have previously reported that innate-like γδ T cells are involved in the early lung host defense during pulmonary hookworm infection (30), γδ T cells have sparse C5a receptor expression.

Eosinophils are also considered essential for resistance against helminths (51–54). Transgenic mice that over-express IL-5 display constitutive eosinophilia and are highly resistant to infection with *N. brasiliensis* (55). However, the mechanisms of how eosinophils provide resistance to helminth infection deserve further investigation. It has been suggested that complement activation may be important for eosinophil recruitment to the skin via generation of the anaphylatoxins, C3a and C5a (24). In contrast, during the lung stage of a primary infection, larvae no longer bind complement and eosinophils do not appear to be recruited and/or fail to recognize larvae at this site (24, 56). These findings suggests that eosinophils can limit infection in a complement-independent manner. Here, we show that complement is nonetheless activated locally in lungs with abundant presence of C5a in BALF and lung homogenates, which was associated with a significant influx of C5aR1-expressing eosinophils.

Alternatively activated macrophages intervene in host immune defense against *N. brasiliensis*, by attacking larvae that migrate to the lungs, and by promoting the repair of damaged tissue (47–49, 57). These findings would support the hypothesis that C5aR1^-/-^ alveolar macrophages are more efficient in targeting larvae and acquiring a resolution phenotype, consistent with the lower parasite burden observed in C5aR1^-/-^ mice. While the specific contribution of neutrophils to innate immune resistance to hookworm infection is still unclear, some reports highlight their potential role in parasite clearance within the lungs and in protection against reinfection (23, 37). Despite previous *in vitro* studies showing a limited role of complement in neutrophil attraction towards larvae (23), our data provides *in vivo* evidence of the importance of C5aR1 in the recruitment of neutrophils to the lung. This finding is supported by the evidence that the alternative pathway and C5aR1 (using PMX53 antagonist) recruits neutrophils and eosinophils to the skin of mice within 30 minutes after inoculation of *N. brasiliensis* (24, 26).

Hookworms employ an array of host evasion strategies including release of proteases and protease inhibitors. For example, hookworm larvae degrade NETs by secreting a deoxyribonuclease (DNase II) (23). *N. americanus* is thought to release an IgA protease that cleaves host antibodies to yield Fab fragments, thus preventing efficient classical and lectin pathway activation (6, 58). *N. brasiliensis* larvae can bind the complement inhibitory protein factor H *in vitro* (59). Furthermore, the filarial parasite, *Onchocerca volvulus*, evades human complement attack by direct binding of factor H and by promoting inactivation of C3b (60). A limited number of studies have investigated the role of complement C3 and C5 *in vivo* during other helminth infections (*Echinococcus granulosus, Schistosoma mansoni*, or *Strongyloides stercoralis*), and have reported no effect or opposite effects in parasite burden when C3 or C5 were depleted or knocked out (61–64). More studies would need to be conducted to disentangle the pleiotropic effects of various complement components and anaphylatoxins during different phases of various types of helminth infection.

Altogether, our findings show that complement is critically involved in the host response to hookworm infection by promoting the recruitment of leukocytes to infected tissues and by modulating the function of cytotoxic effector leukocytes. In the future, a better in-depth understanding of the role of complement activation during hookworm infection is fundamental in deciphering the mechanisms of immune defense. This holds significant implications for the development of therapeutics and vaccines targeting hookworm infection in humans with this neglected tropical disease.

## Data Availability

The datasets presented in this study can be found in online repositories. The names of the repository/repositories and accession number(s) can be found below : GSE272613 (GEO).
