# Supplementary material for: Disruption of the C5a/C5aR1 axis confers protection against hookworm infection in lung
Source: Front Immunol. 2024 Nov 19;15:1475165. doi: 10.3389/fimmu.2024.1475165 (PMC11611822; doi:10.3389/fimmu.2024.1475165)
Supplement: Supplementary file 1 [file DataSheet1.docx]

Supplementary Material

# Supplementary RNA-seq Method

RNA was extracted using QIAGEN RNAeasy Plus micro kit. Ultra-low input RNA-seq was performed by Novogene (USA). The paired-end FASTQ files (150 bp) from Novogene were aligned to the reference mouse genome (GRCm39 Gencode vM32) using the STAR (v2.7.10b) aligner (1), and the samtools (v1.12) software (2) was used to the index and sort the aligned reads. The featureCounts module from the subread (v2.0.3) package (3) was used for quantification. The DESeq2 (v1.38.3) package (4) in R (v4.2.3) (5) run through the RStudioServer interface (6) was used for exploratory and differential expression (DE) analyses. After variance standardized transformation (vst), the counts data were used for visualization of the principal component analysis (PCA) plot through the pcaExplorer (v2.24.0) package (7). One outlier sample among the wild type (WT) control mice that clustered separately in the PCA plot was excluded from further analysis. DE analysis was run with DESeq2 using Wald’s test and Benjamini-Hochberg (BH) multiple testing method. The shrinkage of the log2FoldChange estimates for data visualization was performed using the apeglm (v1.23.1) package (8). DE genes with adjusted P<0.05 were considered statistically significant. Annotation of the DE results were performed through the biomaRt (v2.54.0) package (9) based on Ensembl (release 109). Gene set enrichment analysis (GSEA) was performed using clusterProfiler (v4.6.2) (10) based on hallmark gene sets retrieved using msigdbr (v 7.5.1), the R interface to the Molecular Signatures Database. The input for GSEA was a ranked list of genes based on the product of -log10(nominal P value) and the sign of log2 fold change from the DE results. Multiple test correction for GSEA was based on the BH method with threshold for statistical significance set at adjusted P <0.05. Heatmaps were generated using the vst counts through the ComplexHeatmap (v2.14.0) package (11) and all other figures were generated using ggplot2 (v3.4.1) (12).

**References**

1. Dobin A, Davis CA, Schlesinger F, Drenkow J, Zaleski C, Jha S, et al. STAR: ultrafast universal RNA-seq aligner. Bioinformatics. 2013;29(1):15-21.

2. Danecek P, Bonfield JK, Liddle J, Marshall J, Ohan V, Pollard MO, et al. Twelve years of SAMtools and BCFtools. Gigascience. 2021;10(2).

3. Liao Y, Smyth GK, Shi W. featureCounts: an efficient general purpose program for assigning sequence reads to genomic features. Bioinformatics. 2014;30(7):923-30.

4. Love MI, Huber W, Anders S. Moderated estimation of fold change and dispersion for RNA-seq data with DESeq2. Genome Biology. 2014;15(12).

5. R Core Team. R: A language and environment for statistical computing. R Foundation for Statistical Computing, Vienna, Austria. 2023.

6. RStudio Team. RStudio: Integrated Development Environment for R. RStudio, PBC, Boston, MA. 2023.

7. Marini F, Binder H. pcaExplorer: an R/Bioconductor package for interacting with RNA-seq principal components. BMC Bioinformatics. 2019;20(1):331.

8. Zhu A, Ibrahim JG, Love MI. Heavy-tailed prior distributions for sequence count data: removing the noise and preserving large differences. Bioinformatics. 2019;35(12):2084-92.

9. Durinck S, Spellman PT, Birney E, Huber W. Mapping identifiers for the integration of genomic datasets with the R/Bioconductor package biomaRt. Nature Protocols. 2009;4(8):1184-91.

10. Wu T, Hu E, Xu S, Chen M, Guo P, Dai Z, et al. clusterProfiler 4.0: A universal enrichment tool for interpreting omics data. The Innovation. 2021;2(3):100141.

11. Gu Z, Eils R, Schlesner M. Complex heatmaps reveal patterns and correlations in multidimensional genomic data. Bioinformatics. 2016;32(18):2847-9.

12. Wickham H. ggplot2: Elegant Graphics for Data Analysis. Use R! 2016.

# Supplementary Figures and Tables

## Supplementary Movie

**Movie 1.** Visualization of *N. brasiliensis* L3 infective larvae by binocular microscopy.

Hookworms (~ 3-6 mm) were observed with a magnification of x40 under a binocular microscope.

## Supplementary Figures


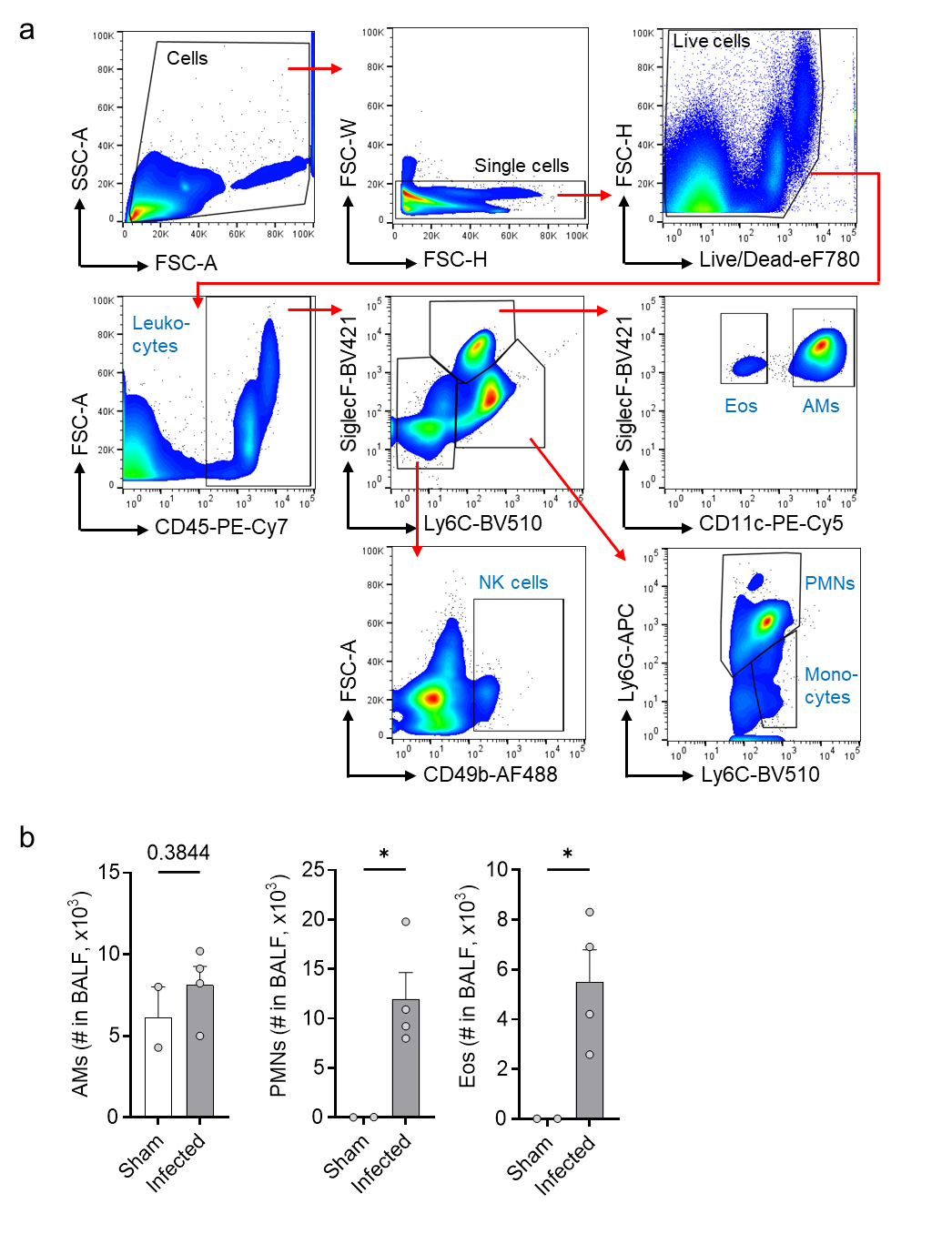


**Supplementary Figure 1.** Flow cytometry analysis of BALF cells after primary infection with *N. brasiliensis*. (a) C5aR1^-/-^ and C57BL/6J wild-type (WT) mice were primarily infected with *N. brasiliensis* (n=500 L3 per mouse, s.c.) and a single-cell suspension of bronchoalveolar lavage cells was collected after 48 h. BALF cells were maintained on ice, separated from supernatants, and analyzed by flow cytometry according to the gating strategy presented. Dead cells positive for Live/Dead eF780 are off-scale and cluster on the right boundary of the plot showing the live cells gate. Backgating revealed that the populations emitting background signals for Live/Dead-eF780 at approximately x=5*10^3^ and x=10^3^ are AMs and a mix of other immune cells, respectively. (b) Absolute numbers (#) of SiglecF^+^CD11c^+^ AMs, Ly6G^+^Ly6C^+^ PMNs and SiglecF^+^CD11c^-^ Eos isolated from the BALF of WT mice that were infected for 42 hours (n=4) or injected s.c. with PBS (Sham, n=2) were determined by flow cytometry. Data are representative of 2 independent experiments. PMNs, polymorphonuclear leukocytes or neutrophils. AMs, alveolar macrophages. Eos, eosinophils. NK, natural killer cells. BALF, bronchoalveolar lavage fluid.


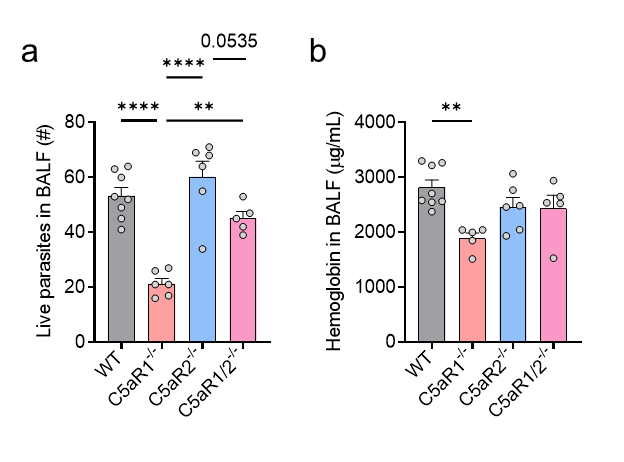


**Supplementary Figure 2.** Effect of C5aR1 and C5aR2 signaling on lung injury and alveolar parasite burden after primary infection with *N. brasiliensis*. C5aR1^-/-^, C5aR2^-/-^, C5aR1/2^-/-^ and WT mice were primarily infected with *N. brasiliensis* (n=500 L3 per mouse, s.c.) and BALF was collected after 48 h. (a) Parasite burden in BALF presented as the worm count (#). (b) Hemoglobin reflecting alveolar hemorrhage and lung injury in BALF. Each circle represents an individual mouse (n≥5/group). Data are presented as mean ± SEM and were analyzed by ordinary one-way ANOVA with Tukey’s multiple comparisons test.*P<0.05; **P<0.01; ***P<0.001, ****P<0.0001. BALF, bronchoalveolar lavage fluid.

**
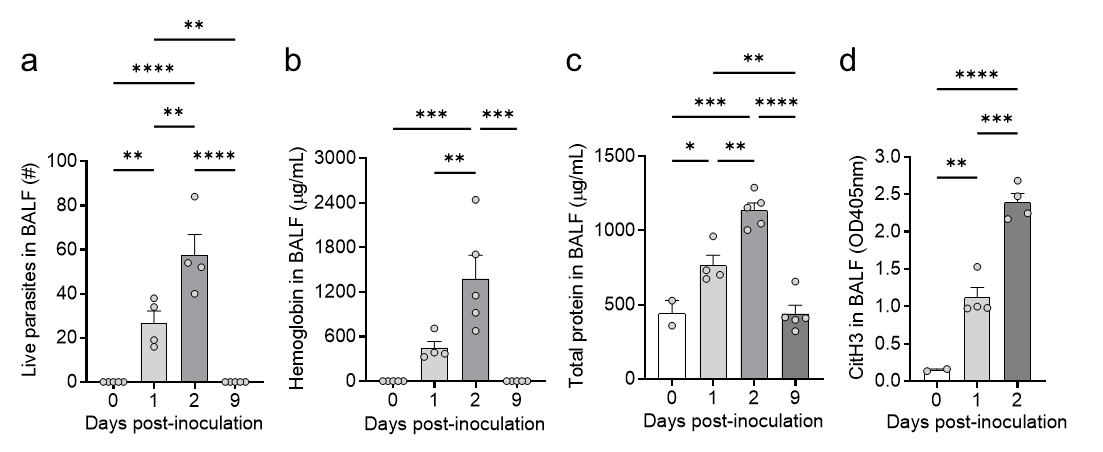
**

**Supplementary Figure 3.** *N. brasiliensis* infection induces lung injury. BALF from WT mice was harvested on days 1, 2 and 9 after infection with *N. brasiliensis* (n=500 L3 per mouse, s.c.). Sham mice (PBS, s.c.) were included as controls. (a) Parasite burden in BALF. # represents the absolute number of worms (n≥4/group). (b) Hemoglobin as a marker of alveolar hemorrhage was quantified in fresh BALF samples by colorimetric assay (n≥4/group). (c) Total protein concentration, a measure of alveolar-capillary barrier disruption, was determined using a BCA assay (n≥4/group except in sham group were n=2). (d) CitH3, a marker of NETosis (n≥4/group except in sham group were n=2). Data are representative of 2 independent experiments. Each circle represents an individual mouse. Data are shown as mean ± SEM and were analyzed by one-way ANOVA with Tukey’s multiple comparisons test.*P<0.05; **P<0.01; ***P<0.001*.* BALF, bronchoalveolar lavage fluid. CitH3, citrullinated histone 3.

**
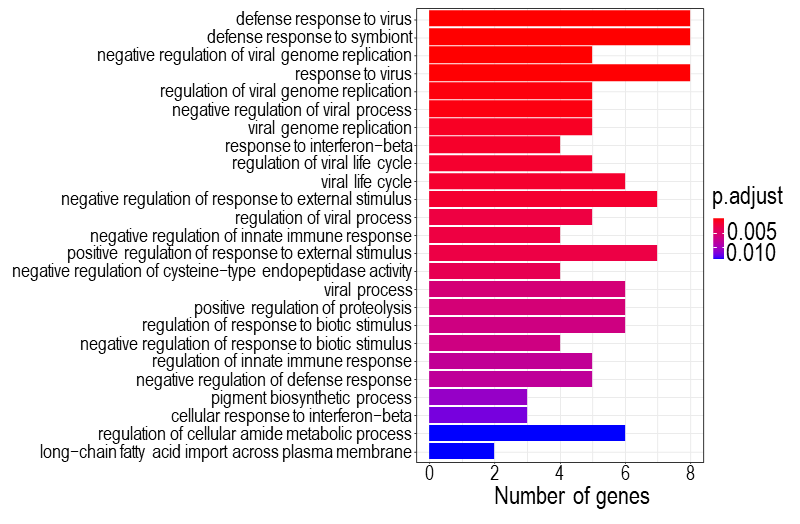
**

**Supplementary Figure 4.** Functional enrichment in C5aR1^-/-^ neutrophil transcriptome during *N. brasiliensis* infection. BALF from C5aR1^-/-^ and WT mice was harvested 48 h after infection with *N. brasiliensis* (n=500 L3 per mouse, s.c.). Alveolar Ly6G^+^Ly6C^+^ PMNs were FACSorted, lysed and processed for bulk RNA sequencing. The bar plot shows significant gene ontology biological processes from the enrichment analysis. DEG with a baseMean greater than 25 and adjusted P<0.05 were included in the analysis. Genes related to hemoglobin processes were excluded. DEG, differentially expressed gene. BALF, bronchoalveolar lavage fluid. PMNs, polymorphonuclear leukocytes or neutrophils.

## Supplementary Table

| Flow cytometry antibodies | | | | |
| --- | --- | --- | --- | --- |
| Reagent or Resource | **Clone** | **Dilution** | **Source** | **RRID** |
| AF488 rat anti-mouse CD49b | DX5 | 1:200 | BioLegend | AB_492879 |
| AF488 rat IgM, k isotype control | RTK2118 | 1:200 | BioLegend | AB_1659271 |
| PE-Cy7 rat anti-mouse CD45 | 30-F11 | 1:200 | eBioscience | AB_2734986 |
| PE-Cy7 rat IgG2b κ isotype control | RTK4530 | 1:200 | Biolegend | AB_326560 |
| APC rat anti-mouse Ly6G | 1A8 | 1:400 | BioLegend | AB_2227348 |
| APC rat IgG2a κ isotype control | RT2758 | 1:400 | BioLegend | AB_2814702 |
| PE-Cy5 Armenian hamster anti-mouse CD11c | N418 | 1:100 | BioLegend | AB_493566 |
| PE-Cy5 Armenian hamster IgG isotype control | HTK888 | 1:100 | BioLegend | AB_326595 |
| BV421 rat anti-mouse SiglecF | E50-2440 | 1:400 | BD Biosciences | AB_2722581 |
| BV421 rat IgG2a κ isotype control | R35-95 | 1:400 | BD Biosciences | AB_11153860 |
| BV510 rat anti-mouse Ly6C | HK1.4 | 1:100 | BioLegend | AB_2562351 |
| PE rat anti-mouse CD88 (C5aR1) | 20/70 | 1:200 | BioLegend | AB_2243735 |
| PE rat IgG2b κ isotype control | RTK4530 | 1:200 | BioLegend | AB_326552 |
| PE rat anti-mouse CCRL2 | BZ2E3 | 1:50 | BD Pharmingen | AB_2739023 |
| PE rat IgG2a κ isotype control | RTK2758 | 1:50 | BioLegend | AB_326530 |
| Unconjugated rabbit anti-mouse DEDD2 | n/a | 1:100 | Bioss | n/a |
| Rabbit IgG isotype control | n/a | 1:100 | Invitrogen | AB_2532938 |
| PE donkey anti-rabbit IgG | Poly4064 | 1:200 | Biolegend | AB_2563484 |
| Unconjugated mouse anti-mouse THBS1 | A6.1 | 1:100 | Invitrogen | AB_10984611 |
| Mouse IgG1 isotype control | Ppv-06 | 1:100 | Invitrogen | AB_2536773 |
| APC goat anti-mouse IgG | Poly4053 | 1:200 | Biolegend | AB_315011 |
| eFluor780 Fixable Viability Dye (FVD) | n/a | 1:1000 | eBioscience | n/a |
| Rat anti-mouse CD16/32 (TruStainFcX) | 93 | 1:50 | BioLegend | AB_1574975 |

| qPCR primers | | |
| --- | --- | --- |
| Primer | **Sense (5’ ➞ 3’)** | **Antisense (5’ ➞ 3’)** |
| Mouse *Gapdh* | TACCCCCAATGTGTCCGTCGTG | CCTTCAGTGGGCCCTCAGATGC |
| Mouse *Dedd2* | CCCCTGAGCGATACAGCTATG | CCTGTGTCCCACTGACTCTGA |
